# Population-level and individual-level explainers for propensity score matching in observational studies

**DOI:** 10.3389/fonc.2022.958907

**Published:** 2022-10-20

**Authors:** Debashis Ghosh, Arya Amini, Bernard L. Jones, Sana D. Karam

**Affiliations:** ^1^ Department of Biostatistics and Informatics, Colorado School of Public Health, Aurora, CO, United States; ^2^ Department of Radiation Oncology, City of Hope Hospital, Los Angeles, CA, United States; ^3^ Department of Radiation Oncology, University of Colorado School of Medicine, Aurora, CO, United States

**Keywords:** propensity score (PS) matching (PSM), exclusion criteria, machine learning, nonrandomized studies, tree based models

## Abstract

**Precis:**

The exclusion of unmatched observations in propensity score matching has implications for the generalizability of causal effects. Machine learning methods can help to identify how the study population differs from the unmatched subpopulation.

**Background:**

There has been widespread use of propensity scores in evaluating the effect of cancer treatments on survival, particularly in administrative databases and cancer registries. A byproduct of certain matching schemes is the exclusion of observations. Borrowing an analogy from clinical trials, one can view these exclusions as subjects that do not satisfy eligibility criteria.

**Methods:**

Developing identification rules for these “data-driven eligibility criteria” in observational studies on both population and individual levels helps to ascertain the population on which causal effects are being made. This article presents a machine learning method to determine the representativeness of causal effects in two different datasets from the National Cancer Database.

**Results:**

Decision trees reveal that groups with certain features have a higher probability of inclusion in the study population than older patients. In the first dataset, younger age categories had an inclusion probability of at least 0.90 in all models, while the probability for the older category ranged from 0.47 to 0.65. Most trees split once more on an even higher age at a lower node, suggesting that the oldest patients are the least likely to be matched. In the second set of data, both age and surgery status were associated with inclusion.

**Conclusion:**

The methodology presented in this paper underscores the need to consider exclusions in propensity score matching procedures as well as complementing matching with other propensity score adjustments.

## Introduction

As identified in a recent review article, a major analytical challenge in oncology-based health services research is the development and application of causal inference methods (1). While databases, such as SEER, SEER-Medicare, and other claims-based datasets exist to answer treatment questions in radiation oncology, the lack of randomization presents a significant obstacle. Thus, a critical step in the analysis is to properly adjust for confounders that might obfuscate the association between treatment and outcome. A variety of methods are available to address this issue, including multivariate regression adjustment, instrumental variables, and propensity score modeling; these are further detailed in Jagsi (1). More generally, it is important to understand how to reconcile evidence from the analyses observational studies versus randomized trials in cancer; several authors have shown evidence of discordance between these disparate sources of information (2, 3).

Recently, there has been much work on the use of propensity score methods in the evaluation of treatments in radiation oncology using the databases mentioned previously. The propensity score is defined as the probability of treatment conditional on confounders (4). For binary treatments, the propensity score is typically modeled using logistic regression, although newer methods based on machine learning techniques also exist (5). Given the estimated propensity score, the treatment effect of interest can be estimated using a variety of approaches, including inverse probability weighting, subclassification, regression adjustment, and matching (6).

This article focuses on matching, the premise of which is that for every treated subject, one or more untreated subjects exist with similar propensity scores. Since scores fall on a continuum, the advice of Rosenbaum and Rubin (7) is to match within a certain caliper distance. Typically, a 1:1 matching scheme or more generally, a K:1 matching scheme, where K denotes the number of control subjects, is used in practice. Thus, by definition, observations that do not satisfy the matching criteria are discarded from the dataset.

This article takes the complementary view that unmatched observations represent subjects for whom causal inferences cannot be made based on the observed data. By contrast, matched observations represent what we term treatment-relevant individuals. These are the subjects for whom it is possible to estimate treatment effects. We argue that the study and identification of treatment-relevant individuals is important in its own right for the following reasons:

1. It can identify violations of assumptions typically used in causal inferential analyses;
2. It can target the subpopulation in our data for which it is appropriate to make inferences;

Regarding (2), an underappreciated point in the literature is that the exclusion of subjects during matching effectively defines a data-adaptive causal estimand. This is contrary to how causal effects are typically formulated in the statistical literature, wherein a causal estimand is defined for the entire study population (6). More recently, given the importance of increasing the representation of communities that have been historically under-represented in clinical trials and medical studies, (2) could help to identify gaps in representation.

Additionally, modeling treatment relevancy as a function allows us to see how variables in the original dataset systematically differ from those in the matched dataset. This is performed using recursive-partitioning analysis (RPA) (8), which was initially used in radiation oncology by Gaspar (9) for identification of prognostic factors in brain metastasis clinical trials conducted by the Radiation Therapy Oncology Group, though the goal of RPA here is different.

Our aim is to understand what factors make the matched dataset different from the original dataset not only at the population level, but also at the individual level. Using interpretable machine learning (10), a recently developed procedure from the data mining community, we identify factors that explain treatment relevance on an individual level. We apply the proposed methodology to data previously analyzed by Amini (11) and Rusthoven (12) from the National Cancer Database (NCDB).

## Materials and methods

### Data source and patient selection

The NCDB is a joint project of the Commission on Cancer of the American College of Surgeons and the American Cancer Society. It is a hospital-based registry that represents 70% of all cancer cases in the US, drawing data from more than 1,500 commission-accredited cancer programs. The American College of Surgeons and the Commission on Cancer have not verified and are not responsible for the analytic or statistical methodology employed, or the conclusions drawn from these data by the investigator. The NCDB has established criteria to ensure the data submitted meet specific quality benchmarks. The following NCDB analysis was performed with the approval of our local institutional review board.

**Figure 1:**
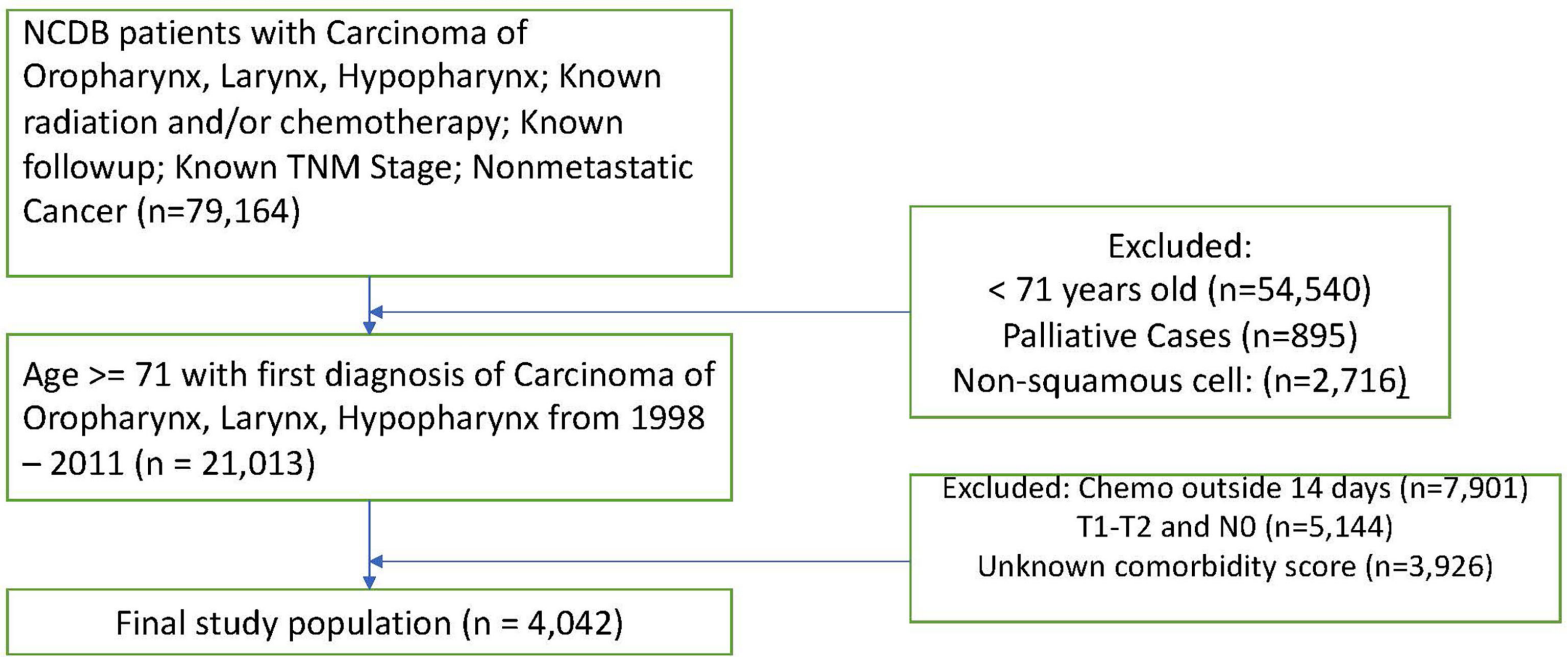
Flow diagram for exclusion criteria in study by Amini et al.

We apply our methodology to two separate NCDB datasets. The first, which was originally analyzed in Amini (11), is comprised of head and neck squamous cell carcinoma patients over 70. With this data, we examine the study population after matching patients on propensity scores for starting chemotherapy (CT) within 14 days of beginning radiation therapy (RT). Scores were calculated given age, sex, race, Charlson-Deyo comorbidity score, tumor stage, nodal stage, and cancer site (oropharynx, larynx, or hypopharynx). Missing data were coded as missing. Table A in the Supplementary Materials section provides descriptive statistics for this study. **Figure 1** shows a flow diagram detailing the exclusion criteria for these data.

The second dataset, first examined in Rusthoven (12), is comprised of patients over the age of 65 with newly diagnosed glioblastoma who underwent one of three treatments – CT, RT, or combined modality therapy (CMT). The NCDB was queried for patients who were 65 years of age with newly diagnosed GBM from 2005 onwards. Exclusions were made for the following reasons: palliative care, deaths within 1 month of diagnosis. Observations with missing data were excluded. Complete data sets for RT, CT, surgery, Charlson-Deyo comorbidity score, age, sex, and year of diagnosis. Molecular data including *MGMT* status were not available for analysis. Subjects were matched on the probability of receiving each treatment compared to each of the other two separately. Propensity scores were calculated given age, sex, race, Charlson-Deyo comorbidity score, year of diagnosis, and an indicator for surgery.

### Statistical analysis

#### Proposed algorithm

We let the T denote the treatment variable and **Z** the vector of confounders. Mathematically, the propensity score (4) is given by


e(Z) = P(T = 1|Z)

Based on the observed data (T_i_,**Z**_i_), i=1,…,n, we typically estimate e(**Z**) using logistic regression. In this article, we used logistic regression in conjunction with several machine learning methods. Once we estimated the propensity scores, a matching algorithm was used to match observations with T = 1 to those with T = 0 with similar values of e(**Z**). This was done using a convex relaxation of the matching problem, which involves formulation of a bipartite graph and network flow optimization algorithms in order to identify matched sets (13). An intuitive overview of propensity score matching with a 1-1 scheme can be found in Box 1.

Box 1 **Explanation of propensity score matching.**

In propensity score matching, the following steps are performed:

1. A propensity score model is fit to all subjects, i=1,…,n. The propensity score fits a regression model with treatment as the outcome model and confounders as the independent variables.

2. Estimate propensity scores for all n subjects.

3. For each subject in the treatment group, the subject in the control group with the closest propensity score is found. The closeness is determined using a caliper metric.(13) Once the control is found, it is removed from the dataset. This is known as 1-1 matching.

4. Step 3 is repeated for each of the treated subjects in the dataset.

5. If there are any treated subjects without controls, they are removed from the dataset.

Note that we can modify step 3 in Box 1 in order to perform variants of 1-1 matching; these include k:1 matching, variable matching, frequency matching and greedy matching. As described by Traskin and Small (14), evaluation of treatment-relevant observations and unmatched subjects has the potential to be informative. Using classification and regression trees (CART) (8), we propose a tree-based approach for modelling the probability of being in the matched set. A tree classifier works as follows: beginning with the initial dataset, it repeatedly splits nodes based on one covariate in **Z**. This continues until the algorithm stops splitting by some stopping criterion. Each terminal node is then assigned a class label by the majority of subjects that fall in that terminal node. Once a test data point with a covariate vector **Z** is introduced, it is run from the top of the tree until it reaches one of the terminal nodes. The class label of the terminal node will then make the prediction. Compared to parametric algorithms, tree-based algorithms have several advantages. First, they tend to be much more flexible than parametrically specified regression models. Second, the algorithm for tree construction only requires criteria for splitting and termination. Third, by splitting a tree based on different covariates at different nodes, the algorithm automatically includes higher-order interaction terms. Finally, they can be nicely visualized.

One major issue in fitting tree-based classifiers is their tendency to overfit. An overfit tree will have strong predictive performance on the original dataset but yield high prediction errors for new data. To solve this problem, Breiman et al. (8) proposed pruned CART in which a tree is fully grown and then pruned back using a cross-validated error criterion. Compared to a standard tree classifier, the pruned tree is smaller in size and yields lower prediction errors on future datasets. Here, we used a cost-complexity measure, implemented in the R package **rpart**, as a criterion on which to determine tree size.

Our algorithm thus consists of the following steps: (a) determine using a matching algorithm which observations are included in the final sample and which are not; denote these observations as “in”/”out” observations; (b) fit a model for the probability of being an “in” observation using the existing covariates with CART; (c) Prune the tree minimizing the cost complexity measure.

We began by estimating propensity scores for the probability that chemotherapy started within 14 days of the RT start date using six methods: logistic regression, **rpart**, random forest, covariance balancing score estimation (CBPS), support vector machines (SVM) and the Toolkit for Weighted Analysis of Nonequivalent Groups (TWANG). The same was applied to the second study (11) with the goal of estimating the probability of receiving each treatment (CMT, RT, or CT) over each of the others individually. We then matched propensity scores using K:1 nearest neighbors with replacement using the **MatchIt** package in R, which employs the nonparametric matching methods suggested by Ho, Imai, King and Stuart (13).

After matching, decision trees were grown for each set of propensity scores with the goal of identifying the features that best explain treatment relevance. Each tree was then pruned using the complexity parameter equal to its minimum cross validated error.

### Population versus individual-level explainers

The proposed methods are capable of detecting variables that correlate with inclusion in the matched sample. We can thus think of this as a population-level explainer. However, it is also important to understand the factors associated with inclusion at a subject-specific level. To construct individual-level explainers, we employed the Local Interpretable Model-Agnostic Explanation (LIME) algorithm (10), which assumes a linear model in the neighborhood of the predicted value. This allows us to gain a better sense of the importance of a given feature in the model’s decision of whether or not an observation was matched. LIME works by predicting n permutations of an observation with the model of interest and then calculates a similarity score for each permutation. Based on the similarity score, LIME selects the features with the most explanatory power and fits a simple model to calculate weights for each feature. Weights are then presented in an interpretable graphic to clarify how the model reached its classification decision.

## Results

### Head and neck cancer data

#### Patient characteristics

A total of 4,042 patients were included in the first dataset: 1,504 (37%) received RT alone and 2,538 (63%) received CMT. The median follow-up occurred at 23 months (range: 2-126 months). Median age of patients undergoing RT alone was 79 years (71-90) vs. 75 years (71-90) for CMT. The majority of CMT cases were larynx (50.4%), followed by oropharynx (35.5%), and hypopharynx (13.1%). More baseline characteristics are available in the supporting material.

#### Population-level explainer

Patient characteristics by treatment relevance are presented in **Table 1**. Among the various propensity score methods, the number of unmatched observations varies from 431 to 650. The mean age for matched observations is consistently lower at 77 years compared to the mean for the unmatched group, which ranges between 82-84.

**Table 1:** Head and neck cancer patient characteristics by match status for given propensity score method.

Characteristic (%) or (mean (sd))	Logistic regression			CART decision tree			Random forest		
	out	in	p	out	in	p	out	in	p
n	585	3457		431	3611		570	3472	
Age	83 (6)	77 (5)	< .001	84 (5)	77 (5)	< .001	82 (6)	77 (5)	< .001
Sex									
Male	337 (57.6)	2390 (69.1)	< .001	260 (6.3)	2467 (68.3)	.001	340 (59.6)	2387 (68.8)	< .001
Female	248 (42.4)	1067 (3.9)		171 (39.7)	1144 (31.7)		230 (4.4)	1085 (31.2)	
Race									
White	462 (79.0)	2989 (86.5)	< .001	373 (86.5)	3078 (85.2)	.722	455 (79.8)	2996 (86.3)	< .001
Black	108 (18.5)	391 (11.3)		48 (11.1)	451 (12.5)		102 (17.9)	397 (11.4)	
Other	15 (2.6)	77 (2.2)		10 (2.3)	82 (2.3)		13 (2.3)	79 (2.3)	
Facility type									
CCP	82 (14.0)	423 (12.2)	.446	62 (14.4)	443 (12.3)	.343	80 (14.0)	425 (12.2)	.385
CCCP	314 (53.7)	1865 (53.9)		234 (54.3)	1945 (53.9)		295 (51.8)	1884 (54.3)	
Academic/research	189 (32.3)	1169 (33.8)		135 (31.3)	1223 (33.9)		195 (34.2)	1163 (33.5)	
Charlson-Deyo comorbidity score									
0	416 (71.1)	2461 (71.2)	.002	313 (72.6)	2564 (71.0)	.528	414 (72.6)	2463 (7.9)	.024
1	100 (17.1)	722 (2.9)		79 (18.3)	743 (2.6)		96 (16.8)	726 (2.9)	
2	69 (11.8)	274 (7.9)		39 (9.0)	304 (8.4)		60 (1.5)	283 (8.2)	
Cancer site									
Oropharynx	137 (23.4)	1183 (34.2)	< .001	94 (21.8)	1226 (34.0)	< .001	137 (24.0)	1183 (34.1)	< .001
Larynx	246 (42.1)	1799 (52.0)		198 (45.9)	1847 (51.1)		253 (44.4)	1792 (51.6)	
Hypopharynx	202 (34.5)	475 (13.7)		139 (32.3)	538 (14.9)		180 (31.6)	497 (14.3)	
Tumor stage									
T1	28 (4.8)	244 (7.1)	< .001	20 (4.6)	252 (7.0)	< .001	26 (4.6)	246 (7.1)	< .001
T2	97 (16.6)	829 (24.0)		60 (13.9)	866 (24.0)		99 (17.4)	827 (23.8)	
T3	254 (43.4)	1539 (44.5)		213 (49.4)	1580 (43.8)		267 (46.8)	1526 (44.0)	
T4	206 (35.2)	845 (24.4)		138 (32.0)	913 (25.3)		178 (31.2)	873 (25.1)	
Nodal stage									
N0	327 (55.9)	1261 (36.5)	< .001	246 (57.1)	1342 (37.2)	< .001	303 (53.2)	1285 (37.0)	< .001
N1	157 (26.8)	810 (23.4)		102 (23.7)	865 (24.0)		149 (26.1)	818 (23.6)	
N2	87 (14.9)	1276 (36.9)		76 (17.6)	1287 (35.6)		97 (17.0)	1266 (36.5)	
N3	14 (2.4)	110 (3.2)		7 (1.6)	117 (3.2)		21 (3.7)	103 (3.0)	
Characteristic (%) or (mean (sd))	CBPS			SVM			TWANG		
	out	in	p	out	in	p	out	in	p
n	578	3464		650	3392		647	3395	
Age	83 (6)	77 (5)	< .001	82 (6)	77 (5)	< .001	83 (6)	77 (5)	< .001
Sex									
Male	320 (55.4)	2407 (69.5)	< .001	374 (57.5)	2353 (69.4)	< .001	383 (59.2)	2344 (69.0)	< .001
Female	258 (44.6)	1057 (3.5)		276 (42.5)	1039 (3.6)		264 (4.8)	1051 (31.0)	
Race									
White	460 (79.6)	2991 (86.3)	< .001	508 (78.2)	2943 (86.8)	< .001	520 (8.4)	2931 (86.3)	< .001
Black	105 (18.2)	394 (11.4)		118 (18.2)	381 (11.2)		106 (16.4)	393 (11.6)	
Other	13 (2.2)	79 (2.3)		24 (3.7)	68 (2.0)		21 (3.2)	71 (2.1)	
Facility type									
CCP	94 (16.3)	411 (11.9)	.007	93 (14.3)	412 (12.1)	.249	102 (15.8)	403 (11.9)	.016
CCCP	287 (49.7)	1892 (54.6)		336 (51.7)	1843 (54.3)		327 (5.5)	1852 (54.6)	
Academic/research	197 (34.1)	1161 (33.5)		221 (34.0)	1137 (33.5)		218 (33.7)	1140 (33.6)	
Charlson-Deyo comorbidity score									
0	394 (68.2)	2483 (71.7)	.001	442 (68.0)	2435 (71.8)	.012	441 (68.2)	2436 (71.8)	.047
1	111 (19.2)	711 (2.5)		134 (2.6)	688 (2.3)		136 (21.0)	686 (2.2)	
2	73 (12.6)	270 (7.8)		74 (11.4)	269 (7.9)		70 (1.8)	273 (8.0)	
Cancer site									
Oropharynx	133 (23.0)	1187 (34.3)	< .001	153 (23.5)	1167 (34.4)	< .001	138 (21.3)	1182 (34.8)	< .001
Larynx	255 (44.1)	1790 (51.7)		274 (42.2)	1771 (52.2)		286 (44.2)	1759 (51.8)	
Hypopharynx	190 (32.9)	487 (14.1)		223 (34.3)	454 (13.4)		223 (34.5)	454 (13.4)	
Tumor stage									
T1	40 (6.9)	232 (6.7)	< .001	33 (5.1)	239 (7.0)	< .001	35 (5.4)	237 (7.0)	< .001
T2	86 (14.9)	840 (24.2)		109 (16.8)	817 (24.1)		114 (17.6)	812 (23.9)	
T3	261 (45.2)	1532 (44.2)		270 (41.5)	1523 (44.9)		283 (43.7)	1510 (44.5)	
T4	191 (33.0)	860 (24.8)		238 (36.6)	813 (24.0)		215 (33.2)	836 (24.6)	
Nodal stage									
N0	317 (54.8)	1271 (36.7)	< .001	347 (53.4)	1241 (36.6)	< .001	341 (52.7)	1247 (36.7)	< .001
N1	153 (26.5)	814 (23.5)		164 (25.2)	803 (23.7)		176 (27.2)	791 (23.3)	
N2	96 (16.6)	1267 (36.6)		121 (18.6)	1242 (36.6)		115 (17.8)	1248 (36.8)	
N3	12 (2.1)	112 (3.2)		18 (2.8)	106 (3.1)		15 (2.3)	109 (3.2)	

CCP, Community cancer program; CCCP; Comprehensive community cancer program; CART, Classification and Regression Trees; CBPS, Covariate balancing propensity scores; SVM, support vector machines; TWANG, Toolkit for Weighting and Analysis of Nonequivalent Groups.

**Figure 2:**
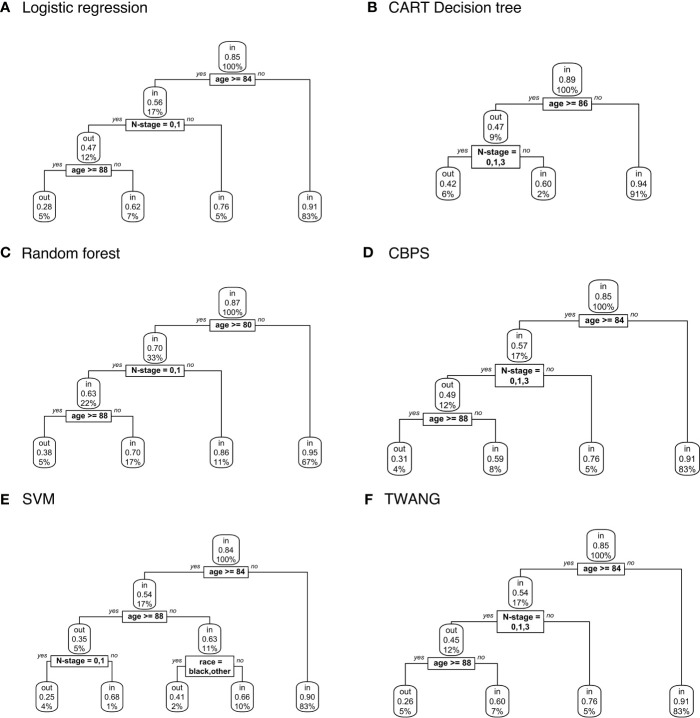
Population-level explainer decision trees for head and neck cancer data classify subjects as matched (“in”) or unmatched (“out”) given observed features using propensity scores estimated by **(A)** logistic regression; **(B)** CART decision tree; **(C)** random forest; **(D)** covariate balancing propensity scores; **(E)** support vector machines; and **(F)** the Toolkit for Weighting and Analysis of Nonequivalent Groups. Each node lists the probability of the corresponding classification for the set of features listed above it and the percentage of subjects that exhibit that set.

**Figure 2** displays decision trees for each method. Pruned trees did not differ from the originals except in the cases of CBPS and TWANG. The results strongly suggest that younger patients are more likely to be included in the study population. Across all trees, the first (and most explanatory) nodes split at ages 80 and above. Although most trees assign the older group a chance of inclusion of greater than 0.5, the probability of this group being matched is still consistently lower than that of the younger group with probabilities of 0.9 and above. The inclusion probability continues to lessen as age increases, which can be seen from the fact that most trees split a second time at age 88, often after accounting for other factors.

#### Subject-specific explainer

**Figure 3:**
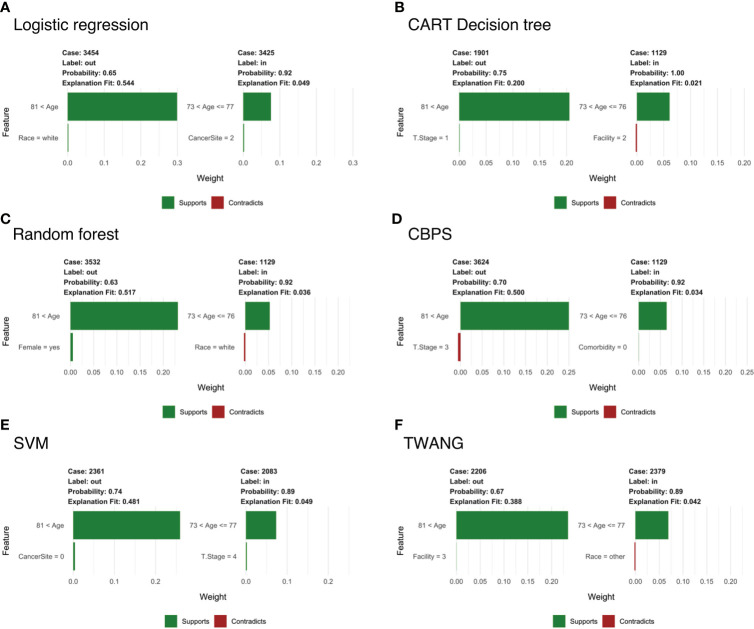
Subject-level Local Interpretable Model-Agnostic Explanations for head and neck cancer data for propensity scores estimated by **(A)** logistic regression; **(B)** CART decision tree; **(C)** random forest; **(D)** covariate balancing propensity scores; **(E)** support vector machines; and **(F)** the Toolkit for Weighting and Analysis of Nonequivalent Groups. Each graphic identifies the most deterministic individual-level features in classifying matched (“in”) or unmatched (“out”) status and notes whether this trait supports or contradicts the probability ascribed by the decision tree. Weights indicate how strongly representative each feature is of the classification.

To examine the specific features that contribute to the predicted outcome for each subject, **Figure 3** compares LIME explanations for matched and unmatched individuals that were correctly classified from each tree. LIME further highlights the importance of age in the classification decision, picking out “81< age” as one of the more representative features for going unmatched. Likewise, it labels age categories between 73-77 as associated with being matched. For example, **Figure 3A** shows case 3454, who is 90 years old and unmatched, while matched case 3425 is 77. For both, age is identified as the feature most responsible for the tree’s prediction, while little weight is assigned to other features.

Notably, LIME’s explanation fit (the R-squared of the simple model fit with the similarity-weighted permuted data) is low for the matched cases listed in **Figure 3**. This pattern continues across all observations and methods with an average explanation fit of 0.16 for unmatched and 0.55 for matched. In this scenario, LIME appears to perform fairly well at describing why patients are excluded, but does less well on predictions for treatment relevance. Likewise, weights of the most representative features are higher in unmatched cases with mean 0.29 across methods compared to matched cases with mean weight 0.06. Alternative analyses can be found in the **Supplementary Information**.

### Glioblastoma data

#### Patient characteristics

This analysis focuses on 11,146 patients with newly diagnosed glioblastoma from a study by Rusthoven (12). A total of 8,435 patients (76%) underwent CMT, 1,693 (15%) received RT alone, and 1,018 (9%) received CT. Using the same techniques employed previously, we estimated the probability of receiving CT alone over RT, CT alone over CMT, and RT alone over CMT. Again, pruned CART trees were grown for each method to identify characteristics that explain treatment relevance.

**Table 2** shows patient characteristics by treatment relevance for logistic regression propensity scores only. Similar results with other algorithms are available in Tables B and C of the Supporting Information. The number of excluded subjects varies widely between the three datasets, ranging from 963 to 7,473. Matched and unmatched subjects differ in almost every category. In particular, the majority of matched cases in the CT vs. RT set have had surgery, while the majority of those matched in RT vs. CMT have not. Also, those in the 80+ age category make up a large proportion of the unmatched group in CT vs. RT (38.1%), while making up a large portion of the matched group in RT vs. CMT (29.4%). Further, white patients are the only group more likely than not to be matched in CT vs. RT whereas the opposite is true in RT vs. CMT.

**Table 2:** Glioblastoma patient characteristics by treatment and match status based on logistic regression propensity scores.

Characteristic (%)	CT vs. RT			CT vs. CMT			RT vs. CMT		
	out	in	p	out	in	p	out	in	p
n	963	1748		7473	1980		6995	3133	
Age									
65-69	152 (15.8)	513 (29.3)	< .001	3085 (41.3)	607 (30.7)	< .001	3041 (43.5)	696 (22.2)	< .001
70-74	215 (22.3)	465 (26.6)		2176 (29.1)	544 (27.5)		2085 (29.8)	761 (24.3)	
75-79	229 (23.8)	424 (24.3)		1390 (18.6)	473 (23.9)		1273 (18.2)	755 (24.1)	
80+	367 (38.1)	346 (19.8)		822 (11.0)	356 (18.0)		596 (8.5)	921 (29.4)	
Sex									
Female	450 (46.7)	934 (53.4)	.001	4290 (57.4)	1064 (53.7)	.004	4083 (58.4)	1565 (50.0)	< .001
Male	513 (53.3)	814 (46.6)		3183 (42.6)	916 (46.3)		2912 (41.6)	1568 (50.0)	
Race									
White	840 (87.2)	1646 (94.2)	< .001	6988 (93.5)	1859 (93.9)	.716	6568 (93.9)	2857 (91.2)	< .001
Black	75 (7.8)	57 (3.3)		278 (3.7)	66 (3.3)		241 (3.4)	167 (5.3)	
Other/unreported	48 (5.0)	45 (2.6)		207 (2.8)	55 (2.8)		186 (2.7)	109 (3.5)	
Charlson-Deyo comorbidity score									
0	640 (66.5)	1133 (64.8)	.64	5241 (70.1)	1295 (65.4)	< .001	4913 (70.2)	2068 (66.0)	< .001
1	180 (18.7)	351 (20.1)		1460 (19.5)	391 (19.7)		1369 (19.6)	603 (19.2)	
2	143 (14.8)	264 (15.1)		772 (10.3)	294 (14.8)		713 (10.2)	462 (14.7)	
Year diagnosed									
2005-2008	628 (65.2)	962 (55.0)	< .001	3796 (50.8)	1057 (53.4)	.043	3427 (49.0)	1924 (61.4)	< .001
2009-2011	335 (34.8)	786 (45.0)		3677 (49.2)	923 (46.6)		3568 (51.0)	1209 (38.6)	
Surgery									
No	467 (48.5)	448 (25.6)	< .001	1695 (22.7)	463 (23.4)	.528	1395 (19.9)	1192 (38.0)	< .001
Yes	496 (51.5)	1300 (74.4)		5778 (77.3)	1517 (76.6)		5600 (80.1)	1941 (62.0)	

CT, chemotherapy; RT, radiation therapy; CMT, combined-modality therapy (RT and CT).

#### Population explainer

**Figure 4:**
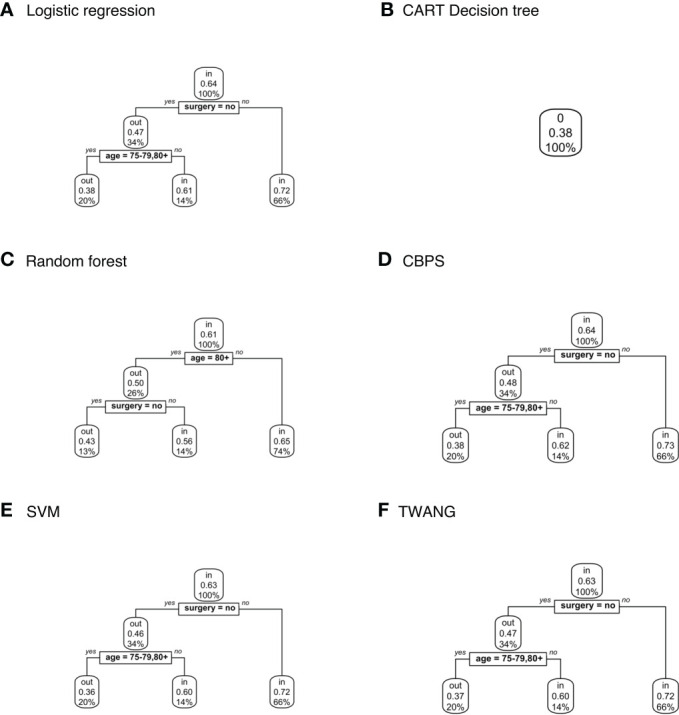
Population-level explainer decision trees for glioblastoma data comparing chemotherapy vs. radiation therapy outcomes. Trees classify subjects as matched (“in”) or unmatched (“out”) given observed features using propensity scores estimated by **(A)** logistic regression; **(B)** CART decision tree; **(C)** random forest; **(D)** covariate balancing propensity scores; **(E)** support vector machines; and **(F)** the Toolkit for Weighting and Analysis of Nonequivalent Groups. Each node lists the probability of the corresponding classification for the set of features listed above it and the percentage of subjects that exhibit that set.

Decision trees classifying treatment relevance can be found in **Figure 4**. Trees for CT vs. RT suggest that those that underwent surgery were more likely to be included with probabilities ranging between 0.65-0.73 than those that did not with probabilities 0.46-0.48. The trees also classify patients 75+ as somewhat less likely to be included with probabilities 0.36-0.38 versus 0.6-0.65 for younger patients.

No tree attempting to explain treatment relevance for the CT vs. CMT set grew for any propensity score method. In other words, the trees assigned the same probability of inclusion in the study population (0.21) to all subjects based on the observed characteristics. Additionally, CART estimated the same propensity score for all subjects within sets – 0.38 for CT vs. RT; 0.11 for CT vs. CMT; and 0.17 for RT vs. CMT. Hence the explainer is inapplicable for these scores.

**Figure 5:**
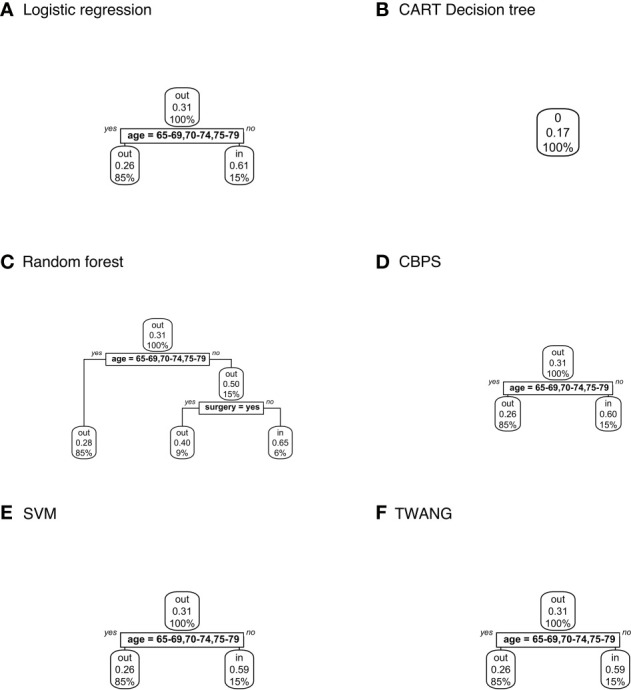
Population-level explainer decision trees for glioblastoma data comparing radiation vs. combined modality therapy. Trees classify subjects as matched (“in”) or unmatched (“out”) given observed features using propensity scores estimated by **(A)** logistic regression; **(B)** CART decision tree; **(C)** random forest; **(D)** covariate balancing propensity scores; **(E)** support vector machines; and **(F)** the Toolkit for Weighting and Analysis of Nonequivalent Groups. Each node lists the probability of the corresponding classification for the set of features listed above it and the percentage of subjects that exhibit that set.

Finally, **Figure 5** shows decision trees from RT vs. CMT data. The majority of these trees classified the 80+ age category as more likely to be included with probability 0.59-0.6 compared to younger ages at 0.26-0.28. The tree modeling CBPS for this set also assigns patients in the 80+ age category who have not had surgery with probability 0.59, compared to 0.26 for younger ages.

#### Subject-specific explainer

**Figure 6:**
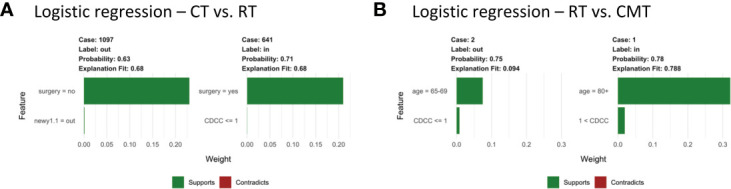
Subject-level Local Interpretable Model-Agnostic Explanations for propensity scores estimated by logistic regression from glioblastoma data comparing both **(A)** chemotherapy vs. radiation and **(B)** radiation vs. combined modality therapy. Each graphic identifies the most deterministic individual-level features in classifying matched (“in”) or unmatched (“out”) status and notes whether this trait supports or contradicts the probability ascribed by the decision tree. Weights indicate how strongly representative each feature is of the classification.

**Figure 6** compares LIME explanations for matched and unmatched observations that were correctly classified from logistic regression propensity score only. The remaining subject-level explanations can be found in supporting material. LIME identifies no surgery as being one of the most deterministic features of going unmatched for the CT vs. RT outcome, while having had surgery was the feature that most contributed to the classification of being matched. The explanation also identifies a Charlson-Deyo comorbidity score of ≤1 as indicative of exclusion, though the weight is comparatively much less than the influence of surgery. For RT vs. CMT, LIME determines age to be the most representative feature for individual-level explanations. Being in a younger age category is one of the most predictive features of going unmatched, while the opposite is true for subjects 80+.

In CT vs. RT, the difference in explanation fits between matched and unmatched are not as large as that in the first study. The average explanation fit across methods for matched subjects is 0.85 versus 0.86 for unmatched. The RT vs. CMT set, however, has and mean explanation fit across methods of 0.83 for matched and 0.11 for unmatched. Weights follow a similar pattern with mean weights 0.24 and 0.29 for matched and unmatched respectively in the CT vs. RT set and 0.31 and 0.06 in RT vs. CMT. Alternative analyses can be found in the **Supplementary Information**.

## Discussion

In this paper, we have proposed a new methodology for interpreting the results from matching algorithms in which observations are discarded from the sample. While there is a lot of work on how to estimate causal effects using matching methods, we view our methodology as a `meta-modeling’ strategy, which has not explored in the literature, with the exception of the of Traskin and Small (14). The focus is not on estimating a causal effect using the matched sample but rather in understanding what factors contribute to inclusion in the matched sample itself. In addition, we take the view that matching is a `black-box’ algorithm whose decisions about matching are unlocked using explainable machine learning. We now point some limitations in the methodology. First, if the proportion of observations in the treatment group represents a small fraction of the total population, then much of the control population will be discarded. This will lead to explainable machine learning models that are built on imbalanced data, which can pose problems in practice. Our advice in those situations is to eschew 1:1 matching in favor of alternative matching schemes, such as k:1 matching or optimal matching. Another limitation of the methodology is the fact that the explainable machine learning models are only as good as the quality of the input variables used in the analysis. A tree-based methodology was used for population-level explanations, which offered several advantages, two of which are incorporation of interactions and intuitive visualization.

The exclusion of unmatched observations in propensity score matching may have consequences beyond just loss of information. Within datasets in this analysis, decision trees consistently identified particular features as more likely to be matched across multiple estimation methods, including age groups and surgery status. By applying these results to the population without regard to age or prior treatment, researchers make the assumption that there is no difference in treatment response between older and younger patients or patients who have and have not had surgery. If these groups of patients tend to respond to treatment differently, the results may not be applicable and therefore beneficial to the subgroup less likely to be included. With the current emphasis on understanding health disparities in vulnerable populations, it is precisely these groups for which matching might be difficult so that they get excluded from matching procedure.

Our results raise a few questions for potential further study. First, when all subjects are given the same probability of inclusion, as with the CART-generated propensity scores for the Rusthoven data, what are the implications for generalizability? How does this scenario compare to the representativeness of the Amini data in which age was clearly correlated with being unmatched, but treatment relevance could not be so easily predicted? Determining the specific outcomes that imply generalizability or lack thereof is necessary to guide the application of this method.

We have argued that describing the subpopulation of treatment relevant individuals is an important stage in propensity score matching. If the matched population is assumed to reflect the population as a whole, inference could be unintentionally generalized to a subpopulation that was not actually analyzed. Ignoring this step is akin to extrapolating the results of a randomized trial to individuals that did not meet the inclusion criteria.

Finally, this study represents a novel application of explainable machine learning methods, which have recently received much attention in the data mining community. Our philosophy has been to view matching as a `black-box’ modeling akin to deep learning and to then use explainable machine learning to better understand the decisions on what subjects get matched.

Note: R code for the methods discussed in this paper is available on GitHub: https://github.com/vzste/ExplainerCode.git

## Data availability statement

The data can be obtained from SK and BJ provided that the requestor give a description about the intended use of the dataset. Requests to access these datasets should be directed to SK, sana.karam@cuanschutz.edu; BJ, Bernard.jones@cuanschutz.edu.

## Ethics statement

Ethical review and approval were not required for the study on human participants in accordance with the local legislation and institutional requirements. Written informed consent for participation was not required for this study in accordance with the national legislation and the institutional requirements.

## Author contributions

Conceptualization: DG. Methodology: DG. Software: DG. Formal analysis: DG. Investigation: AA, BJ, SK, and DG. Resources: AA, BJ, SK, and DG. Data curation: AA, BJ, SK, and DG. Writing – original draft: DG. Writing – review and editing: AA, BJ, SK, and DG. Supervision: SK and DG. Project administration: DG. Funding acquisition: DG. All authors contributed to the article and approved the submitted version.

## Funding

This research is supported by a pilot grant from the Data Science to Patient Value (D2V) initiative from the University of Colorado.

DG would like to acknowledge the following grants: NCI R01 CA129102, NSF DMS 1914937, NSF SES 2149492. SK would like to acknowledge the following grants: NIDCR R01 DE028529-01, NIDCR R01 DE028282-01, and NIDCR/NCI 1P50CA261605-01.

## Conflict of interest

The authors declare that the research was conducted in the absence of any commercial or financial relationships that could be construed as a potential conflict of interest.

## Publisher’s note

All claims expressed in this article are solely those of the authors and do not necessarily represent those of their affiliated organizations, or those of the publisher, the editors and the reviewers. Any product that may be evaluated in this article, or claim that may be made by its manufacturer, is not guaranteed or endorsed by the publisher.
