# Supplementary material for: Population-level and individual-level explainers for propensity score matching in observational studies
Source: Front Oncol. 2022 Oct 20;12:958907. doi: 10.3389/fonc.2022.958907 (PMC9630947; doi:10.3389/fonc.2022.958907)
Supplement: Supplementary file 1 [file DataSheet_1.pdf]

Supporting material

Tables

Table A. Head and neck cancer patient characteristics by treatment				
Characteristic (%) or (mean (sd))	All patients	Radiation alone	Chemoradiation	p
n	4042	1504	2538	
Age	78 (5)	80 (6)	76 (5)	<.001
Sex				
Male	2727 (67.5)	957 (63.6)	1770 (69.7)	<.001
Female	1315 (32.5)	547 (36.4)	768 (30.3)	
Race (%)				
White	3422 (84.7)	1236 (82.2)	2186 (86.1)	.004
Black	498 (12.3)	222 (14.8)	276 (10.9)	
Other	91 (2.3)	34 (2.3)	57 (2.2)	
Missing	31 (.8)	12 (.8)	19 (.7)	
Facility type				
Community cancer program	505 (12.5)	198 (13.2)	307 (12.1)	.607
Comprehensive community cancer program	2179 (53.9)	803 (53.4)	1376 (54.2)	
Academic/research	1358 (33.6)	503 (33.4)	855 (33.7)	
Charlson-Deyo comorbidity score				
0	2877 (71.2)	1067 (70.9)	1810 (71.3)	.035
1	822 (20.3)	289 (19.2)	533 (21.0)	
≥2	343 (8.5)	148 (9.8)	195 (7.7)	
Cancer site				
Oropharynx	1320 (32.7)	418 (27.8)	902 (35.5)	<.001
Larynx	2045 (50.6)	741 (49.3)	1304 (51.4)	
Hypopharynx	677 (16.7)	345 (22.9)	1332 (13.1)	
Tumor stage				
T1	272 (6.7)	91 (6.1)	181 (7.1)	<.001
T2	926 (22.9)	286 (19.0)	640 (25.2)	
T3	1793 (44.4)	689 (45.8)	1104 (43.5)	
T4	1051 (26.0)	438 (29.1)	613 (24.2)	
Nodal stage				
N0	1588 (39.3)	719 (47.8)	869 (34.2)	<.001
N1	967 (23.9)	371 (24.7)	596 (23.5)	
N2	1363 (33.7)	373 (24.8)	990 (39.0)	
N3	124 (3.1)	41 (2.7)	83 (3.3)	

Table B. Glioblastoma patient characteristics characteristics by treatment					
Characteristic (%)	Total	CMT	CT	RT	p
n	11146	8435	1018	1693	
Age					
65-69	4047 (36.3)	3382 (40.1)	310 (30.5)	355 (21.0)	<.001
70-74	3123 (28.0)	2443 (29.0)	277 (27.2)	403 (23.8)	
75-79	2272 (20.4)	1619 (19.2)	244 (24.0)	409 (24.2)	
80+	1704 (15.3)	991 (11.7)	187 (18.4)	526 (31.1)	
Sex					
Male	6193 (55.6)	4809 (57)	545 (53.5)	839 (49.6)	<.001
Female	4953 (44.4)	3626 (43.0)	473 (46.5)	854 (50.4)	
Race					
White	10379 (93.1)	7893 (93.6)	954 (93.7)	1532 (90.5)	<.001
Black	442 (4.0)	310 (3.7)	34 (3.3)	98 (5.8)	
Other/unreported	325 (2.9)	232 (2.8)	30 (2.9)	63 (3.7)	
Charlson-Deyo comorbidity score					
0	7645 (68.6)	5872 (69.6)	664 (65.2)	1109 (65.5)	<.001
1	2177 (19.5)	1646 (19.5)	205 (20.1)	326 (19.3)	
2+	1324 (11.9)	917 (10.9)	149 (14.6)	258 (15.2)	
Year of diagnosis					
2005-2008	5897 (52.9)	4307 (51.1)	546 (53.6)	1044 (61.7)	<.001
2009-2011	5249 (47.1)	4128 (48.9)	472 (46.4)	649 (38.3)	
Surgery					
No	2830 (25.4)	1915 (22.7)	243 (23.9)	672 (39.7)	<.001
Yes	8316 (74.6)	6520 (77.3)	775 (76.1)	1021 (60.3)	
Abbreviations: CT, chemotherapy; RT, radiation therapy; CMT, combined-modality therapy (RT and CT).					

Table C. Glioblastoma patient characteristics by match status – RT vs. CMT						
	Random forest			CBPS		
Characteristic (%)	out	in	p	out	in	p
n	1035	1676		969	1742	
Age						
65-69	195 (18.8)	470 (28.0)	<.001	155 (16.0)	510 (29.3)	<.001
70-74	230 (22.2)	450 (26.8)		212 (21.9)	468 (26.9)	
75-79	263 (25.4)	390 (23.3)		230 (23.7)	423 (24.3)	
80+	347 (33.5)	366 (21.8)		372 (38.4)	341 (19.6)	
Sex						
Female	506 (48.9)	878 (52.4)	.084	457 (47.2)	927 (53.2)	.003
Male	529 (51.1)	798 (47.6)		512 (52.8)	815 (46.8)	
Race						
White	936 (9.4)	1550 (92.5)	.172	841 (86.8)	1645 (94.4)	<.001
Black	58 (5.6)	74 (4.4)		78 (8.0)	54 (3.1)	
Other/unreported	41 (4.0)	52 (3.1)		50 (5.2)	43 (2.5)	
CDCC						
0	701 (67.7)	1072 (64.0)	0.096	640 (66.0)	1133 (65.0)	.636
1	183 (17.7)	348 (2.8)		192 (19.8)	339 (19.5)	
2	151 (14.6)	256 (15.3)		137 (14.1)	270 (15.5)	
Year diagnosed						
2005-2008	663 (64.1)	927 (55.3)	<.001	636 (65.6)	954 (54.8)	<.001
2009-2011	372 (35.9)	749 (44.7)		333 (34.4)	788 (45.2)	
Surgery						
no	441 (42.6)	474 (28.3)	<.001	477 (49.2)	438 (25.1)	<.001
yes	594 (57.4)	1202 (71.7)		492 (5.8)	1304 (74.9)	

Table C, continued. Glioblastoma patient characteristics by match status – RT vs. CMT						
	SVM			TWANG		
Characteristic (%)	out	in	p	out	in	p
n	996	1715		993	1718	
Age						
65-69	162 (16.3)	503 (29.3)	<.001	165 (16.6)	500 (29.1)	<.001
70-74	212 (21.3)	468 (27.3)		213 (21.5)	467 (27.2)	
75-79	245 (24.6)	408 (23.8)		234 (23.6)	419 (24.4)	
80+	377 (37.9)	336 (19.6)		381 (38.4)	332 (19.3)	
Sex						
Female	465 (46.7)	919 (53.6)	.001	471 (47.4)	913 (53.1)	.005
Male	531 (53.3)	796 (46.4)		522 (52.6)	805 (46.9)	
Race						
White	865 (86.8)	1621 (94.5)	<.001	873 (87.9)	1613 (93.9)	<.001
Black	79 (7.9)	53 (3.1)		73 (7.4)	59 (3.4)	
Other/unreported	52 (5.2)	41 (2.4)		47 (4.7)	46 (2.7)	
CDCC						
0	646 (64.9)	1127 (65.7)	.824	652 (65.7)	1121 (65.3)	.900
1	195 (19.6)	336 (19.6)		196 (19.7)	335 (19.5)	
2	155 (15.6)	252 (14.7)		145 (14.6)	262 (15.3)	
Year diagnosed						
2005-2008	650 (65.3)	940 (54.8)	<.001	641 (64.6)	949 (55.2)	<.001
2009-2011	346 (34.7)	775 (45.2)		352 (35.4)	769 (44.8)	
Surgery						
no	494 (49.6)	421 (24.5)	<.001	489 (49.2)	426 (24.8)	<.001
yes	502 (5.4)	1294 (75.5)		504 (5.8)	1292 (75.2)	

Table B. Glioblastoma patient characteristics by match status – CT vs. CMT						
	Random forest			CBPS		
Characteristic (%)	out	in	p	out	in	p
n	7471	1982		7493	1960	
Age						
65-69	3007 (4.2)	685 (34.6)	<.001	3091 (41.3)	601 (3.7)	<.001
70-74	2176 (29.1)	544 (27.4)		2182 (29.1)	538 (27.4)	
75-79	1427 (19.1)	436 (22.0)		1402 (18.7)	461 (23.5)	
80+	861 (11.5)	317 (16.0)		818 (1.9)	360 (18.4)	
Sex						
Female	4271 (57.2)	1083 (54.6)	.046	4301 (57.4)	1053 (53.7)	.004
Male	3200 (42.8)	899 (45.4)		3192 (42.6)	907 (46.3)	
Race						
White	6985 (93.5)	1862 (93.9)	.565	7006 (93.5)	1841 (93.9)	.751
Black	272 (3.6)	72 (3.6)		278 (3.7)	66 (3.4)	
Other/unreported	214 (2.9)	48 (2.4)		209 (2.8)	53 (2.7)	
CDCC						
0	5206 (69.7)	1330 (67.1)	.082	5254 (7.1)	1282 (65.4)	<.001
1	1441 (19.3)	410 (2.7)		1456 (19.4)	395 (2.2)	
2	824 (11.0)	242 (12.2)		783 (1.4)	283 (14.4)	
Year diagnosed						
2005-2008	3816 (51.1)	1037 (52.3)	.337	3805 (5.8)	1048 (53.5)	0.036
2009-2011	3655 (48.9)	945 (47.7)		3688 (49.2)	912 (46.5)	
Surgery						
no	1692 (22.6)	466 (23.5)	.433	1693 (22.6)	465 (23.7)	0.303
yes	5779 (77.4)	1516 (76.5)		5800 (77.4)	1495 (76.3)	

Table B, continued. Glioblastoma patient characteristics by match status – CT vs. CMT						
	SVM			TWANG		
Characteristic (%)	out	in	p	out	in	p
n	7476	1977		7475	1978	
Age						
65-69	3082 (41.2)	610 (3.9)	<.001	3083 (41.2)	609 (3.8)	<.001
70-74	2180 (29.2)	540 (27.3)		2183 (29.2)	537 (27.1)	
75-79	1397 (18.7)	466 (23.6)		1392 (18.6)	471 (23.8)	
80+	817 (1.9)	361 (18.3)		817 (1.9)	361 (18.3)	
Sex						
Female	4292 (57.4)	1062 (53.7)	.003	4285 (57.3)	1069 (54.0)	.010
Male	3184 (42.6)	915 (46.3)		3190 (42.7)	909 (46.0)	
Race						
White	6986 (93.4)	1861 (94.1)	.400	6985 (93.4)	1862 (94.1)	.397
Black	282 (3.8)	62 (3.1)		282 (3.8)	62 (3.1)	
Other/unreported	208 (2.8)	54 (2.7)		208 (2.8)	54 (2.7)	
CDCC						
0	5235 (7.0)	1301 (65.8)	<.001	5242 (7.1)	1294 (65.4)	<.001
1	1458 (19.5)	393 (19.9)		1457 (19.5)	394 (19.9)	
2	783 (1.5)	283 (14.3)		776 (1.4)	290 (14.7)	
Year diagnosed						
2005-2008	3791 (5.7)	1062 (53.7)	.019	3789 (5.7)	1064 (53.8)	.015
2009-2011	3685 (49.3)	915 (46.3)		3686 (49.3)	914 (46.2)	
Surgery						
no	1692 (22.6)	466 (23.6)	.393	1693 (22.6)	465 (23.5)	.435
yes	5784 (77.4)	1511 (76.4)		5782 (77.4)	1513 (76.5)	

Table C. Glioblastoma patient characteristics by match status -- RT v. CMT						
	Random forest			CBPS		
Characteristic (%)	out	in	p	out	in	p
n	6983	3145		7002	3126	
Age						
65-69	2880 (41.2)	857 (27.2)	<.001	3050 (43.6)	687 (22.0)	<.001
70-74	2039 (29.2)	807 (25.7)		2085 (29.8)	761 (24.3)	
75-79	1335 (19.1)	693 (22.0)		1256 (17.9)	772 (24.7)	
80+	729 (1.4)	788 (25.1)		611 (8.7)	906 (29.0)	
Sex						
Female	4025 (57.6)	1623 (51.6)	<.001	4085 (58.3)	1563 (5.0)	<.001
Male	2958 (42.4)	1522 (48.4)		2917 (41.7)	1563 (5.0)	
Race						
White	6574 (94.1)	2851 (9.7)	<.001	6575 (93.9)	2850 (91.2)	<.001
Black	236 (3.4)	172 (5.5)		240 (3.4)	168 (5.4)	
Other/unreported	173 (2.5)	122 (3.9)		187 (2.7)	108 (3.5)	
CDCC						
0	4874 (69.8)	2107 (67.0)	<.001	4923 (7.3)	2058 (65.8)	<.001
1	1366 (19.6)	606 (19.3)		1373 (19.6)	599 (19.2)	
2	743 (1.6)	432 (13.7)		706 (1.1)	469 (15.0)	
Year diagnosed						
2005-2008	3552 (5.9)	1799 (57.2)	<.001	3431 (49.0)	1920 (61.4)	<.001
2009-2011	3431 (49.1)	1346 (42.8)		3571 (51.0)	1206 (38.6)	
Surgery						
no	1512 (21.7)	1075 (34.2)	<.001	1398 (2.0)	1189 (38.0)	<.001
yes	5471 (78.3)	2070 (65.8)		5604 (8.0)	1937 (62.0)	

Table C, continued. Glioblastoma patient characteristics by match status -- RT v. CMT						
	SVM			TWANG		
Characteristic (%)	out	in	p	out	in	p
n	7021	3107		7019	3109	
Age						
65-69	3046 (43.4)	691 (22.2)	<.001	3048 (43.4)	689 (22.2)	<.001
70-74	2086 (29.7)	760 (24.5)		2071 (29.5)	775 (24.9)	
75-79	1270 (18.1)	758 (24.4)		1271 (18.1)	757 (24.3)	
80+	619 (8.8)	898 (28.9)		629 (9.0)	888 (28.6)	
Sex						
Female	4096 (58.3)	1552 (5.0)	<.001	4101 (58.4)	1547 (49.8)	<.001
Male	2925 (41.7)	1555 (5.0)		2918 (41.6)	1562 (5.2)	
Race						
White	6591 (93.9)	2834 (91.2)	<.001	6594 (93.9)	2831 (91.1)	<.001
Black	242 (3.4)	166 (5.3)		242 (3.4)	166 (5.3)	
Other/unreported	188 (2.7)	107 (3.4)		183 (2.6)	112 (3.6)	
CDCC						
0	4927 (7.2)	2054 (66.1)	<.001	4933 (7.3)	2048 (65.9)	<.001
1	1386 (19.7)	586 (18.9)		1374 (19.6)	598 (19.2)	
2	708 (1.1)	467 (15.0)		712 (1.1)	463 (14.9)	
Year diagnosed						
2005-2008	3445 (49.1)	1906 (61.3)	<.001	3448 (49.1)	1903 (61.2)	<.001
2009-2011	3576 (5.9)	1201 (38.7)		3571 (5.9)	1206 (38.8)	
Surgery						
no	1404 (2.0)	1183 (38.1)	<.001	1417 (2.2)	1170 (37.6)	<.001
yes	5617 (8.0)	1924 (61.9)		5602 (79.8)	1939 (62.4)	

Figures

Figure A

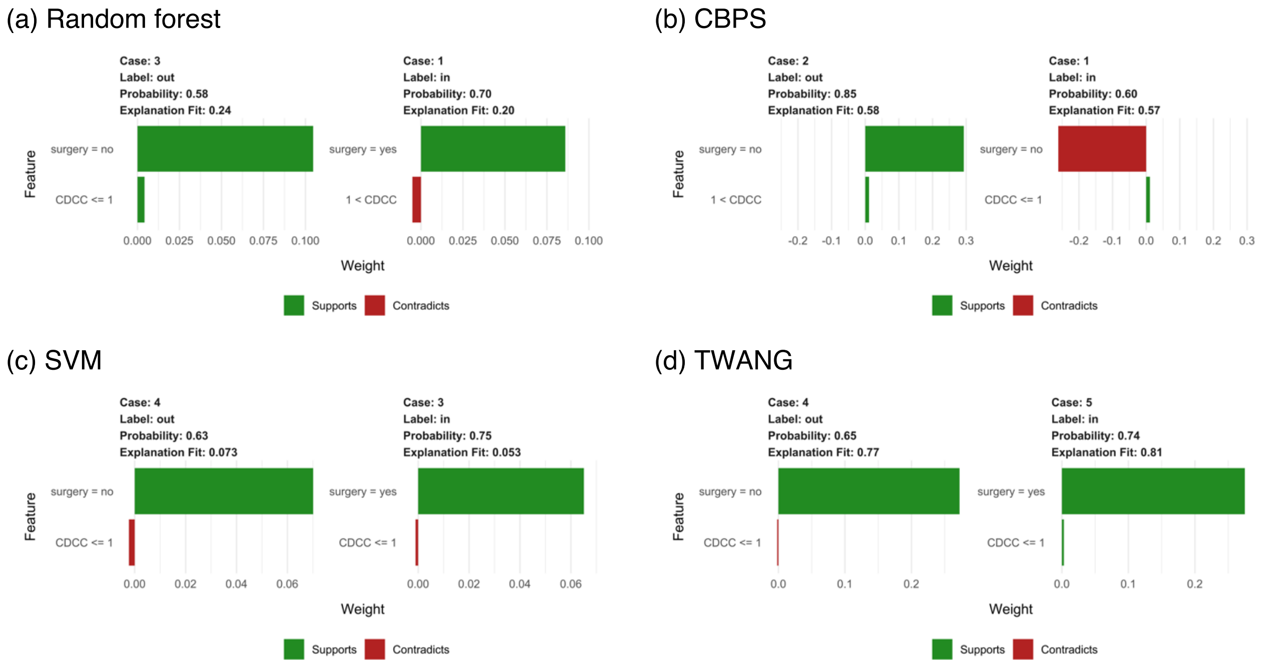

Figure B

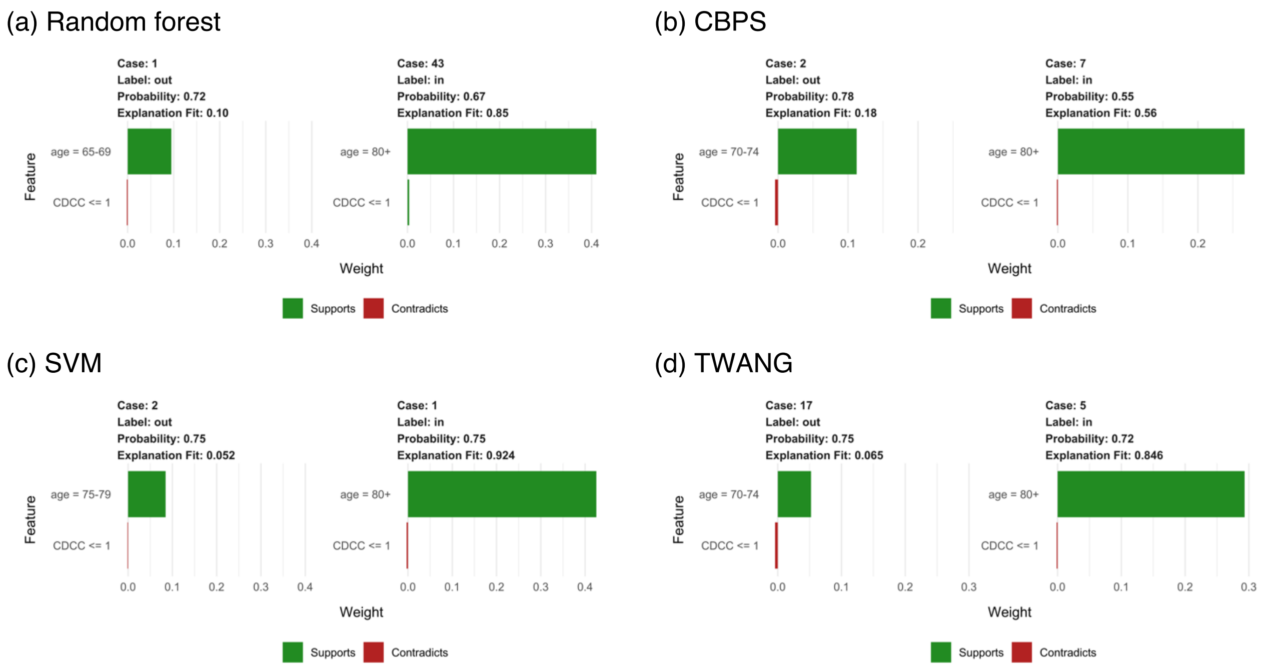

Figure legends

Figures A and B. Subject-level Local Interpretable Model-Agnostic Explanations for propensity scores from glioblastoma data comparing both (A) chemotherapy vs. radiation and (B) chemotherapy vs. combined modality therapy estimated by (a) random forest; (b) covariate balancing propensity scores; (c) support vector machines; and (d) the Toolkit for Weighting and Analysis of Nonequivalent Groups. Each graphic identifies the most deterministic individual-level features in classifying matched ("in") or unmatched ("out") status and notes whether this trait supports or contradicts the probability ascribed by the decision tree. Weights indicate how strongly representative each feature is of the classification.
